# Comparison of Different Keratinocyte Cell Line Models for Analysis of NLRP1 Inflammasome Activation

**DOI:** 10.3390/biom14111427

**Published:** 2024-11-08

**Authors:** Tian Wang, Amir S. Yazdi, Diana Panayotova-Dimitrova

**Affiliations:** Department of Dermatology and Allergology, University Hospital RWTH Aachen, 52074 Aachen, Germany; twang@ukaachen.de (T.W.); ayazdi@ukaachen.de (A.S.Y.)

**Keywords:** keratinocytes, NLRP1 inflammasome, ultraviolet B, talabostat, cytokines

## Abstract

The NLRP1 (nucleotide-binding domain, leucine-rich-containing family, pyrin domain-containing-1) inflammasome is the most important inflammasome in human keratinocytes. It plays a crucial role in regulating innate immunity in the skin. This study aimed to evaluate NLRP1 inflammasome activation and the corresponding levels of detection in different keratinocyte cell lines to identify a suitable in vitro model for analyzing inflammasome activation in keratinocytes. We compared NLRP1 inflammasome activation, expression, and cell death among primary keratinocytes and immortalized keratinocyte cell lines HaCaT, HaSKpw, and SVTERT upon stimulation with ultraviolet B (UVB) irradiation or talabostat. The effects of both NLRP1 inducers on cell death and the modification of NLRP1 molecules were examined using fluorescence-activated cell sorting analysis, Western blotting, and an enzyme-linked immunosorbent assay. The key inflammasome components had varied expression levels among the keratinocyte cell models, with the highest expression observed in primary keratinocytes. Moreover, our data showed that both UVB and talabostat triggered cell death, and NLRP1 inflammasome activation was readily detected in primary keratinocytes but not in the analyzed immortalized keratinocyte cell lines. Therefore, we do not recommend the use of the immortalized keratinocyte cell lines HaCaT, HaSKpw, and SVTERT for analyzing inflammasome activation in keratinocytes; we strongly recommend the use of primary keratinocytes for these studies.

## 1. Introduction

Human skin is the outermost barrier that protects an organism from its surroundings [1]. Keratinocytes are the predominant cell type in the skin epidermis and are organized in a complicated layered structure composed of cells at different stages of differentiation. This organization provides a high level of resilience and durability, which are crucial for defense against chemical and physical damage. Besides its function as a physical barrier, the skin plays a critical role in the immune response, protecting the organism from pathogens [2]. A constant crosstalk between keratinocytes and immune cells present in the skin is required to maintain skin homeostasis and protect the skin from inflammatory diseases. Keratinocytes communicate with immune cells via keratinocyte-derived cytokines and chemokines, which they activate, process, and secrete upon the detection of harm. A proinflammatory feature of keratinocytes is the activation of multimeric protein complexes known as inflammasomes. Upon activation by pathogens or stress signals, intracellular inflammasome complexes known as NOD-like receptors containing a pyrin domain (NLRPs) are formed, leading to caspase-1 activation. Active caspase-1 cleaves its substrates interleukin-1 beta (IL-1β) and interleukine-18 (IL-18) and the pore-forming protein gasdermin D (GSDMD) [3,4]. The N-terminal fragment of GSDMD oligomerizes in the cytoplasmic membrane, forming pores through which IL-1β and IL-18 are released. Moreover, cell swelling and membrane rupture can occur following the formation of GSDMD pores, resulting in pyroptotic cell death [4]. NLRP1 is the main inflammasome sensor and the most prominent inflammasome in the skin [5]. The inducers of the NLRP1 inflammasome include 3C proteases from the human rhinovirus [6], ultraviolet B (UVB) irradiation [7], double-stranded RNA [8], and dipeptidyl peptidase (DPP) 8/DPP9 inhibitors [9]. Abnormal activation or specific mutations in NLRP1 are linked to rare skin diseases, including multiple self-healing palmoplantar carcinomas, keratosis lichenoides chronica, and dipeptidyl peptidase 9 deficiency [10,11,12,13].

Human primary keratinocytes (PKs) isolated from the skin of healthy donors represent the closest system to the in vivo conditions and are therefore the most suitable model for physiological and biochemical studies of the skin. However, the major disadvantages of PKs as an in vitro model are their relatively short life span after isolation and difficulties in culturing and genome editing, which limit their utility for extended studies. In contrast, immortalized human keratinocyte cell lines are easy to culture and manipulate genetically, thus providing stable and reproducible conditions for studying the intracellular signaling pathways that regulate skin biology.

Numerous molecular, cellular, and biochemical methods have been used to evaluate inflammasome activation. A quantitative polymerase chain reaction (qPCR), an enzyme-linked immunosorbent assay (ELISA), and Western blotting are standard techniques for measuring transcriptional or translational changes in inflammasome-related genes and proteins. Western blotting can be used to visualize proteolytic cleavage. Moreover, methods for analyzing cell viability and inflammasome-induced pyroptotic cell death are widely used.

In this study, we analyzed the differences in the activation and detection of the NLRP1 inflammasome upon stimulation with talabostat and UVB in three different immortalized human cell lines, HaCaT, HaSKpw, and NHEK/SVTERT3-5 (SVTERT), as well as in PKs. The levels of activation and detection were determined using Western blotting, ELISA, and cell death analyses. Our results demonstrate that inflammasome activation is robust and reliably and reproducibly detected only in PKs but not in the analyzed immortalized keratinocyte cell lines.

## 2. Materials and Methods

### 2.1. PKs, Cell Lines, and Treatment

Human PKs were isolated and cultured using an established protocol, as previously described [14]. Briefly, PKs were isolated from human skin after removing the adipose tissue. The skin was cut into 2 mm wide strips resembling a comb. The epidermis was separated from the dermis via overnight incubation with 2.4 U/mL Dispase II (Cat# 17105041; Gibco, Waltham, MA, USA) at 4 °C. The next day, the skin was further incubated for 1 to 2 h at 37 °C and the epidermis was peeled off from the dermis and transferred to a plate containing phosphate-buffered saline (PBS). The epidermis was incubated with the solution until the cell suspension was observed under a microscope. Next, the epidermis was incubated in 0.05% Trypsin—0.02% EDTA (Cat# T4174; Sigma-Aldrich, St. Louis, MO, USA) for 5 min at 37 °C, and trypsinization was then stopped via the addition of Dulbecco’s modified eagle medium (DMEM) supplemented with 10% fetal calf serum (FCS). The single-cell suspension was filtered through a 100 μm cell strainer (Cat# 431752; Corning, NY, USA) and centrifuged at 250× *g* for 7 min. Keratinocytes at passage 0 were plated in CnT-PR medium (CELLnTEC) on collagen bovine type I (Cat# 354231; Corning)-coated plastic and cultured until they reached 80% confluency.

All data with PKs shown represent results obtained from independent experiments performed with cells isolated from at least 2 (to 5) different donors.

HaCaT and HaSKpw cells were generously provided by Prof. P. Boukamp, formerly from DKFZ, Heidelberg. Both cell lines were cultured in DMEM supplemented with 10% FCS. NHEK/SVTERT3-5cells were purchased from EVERCYTE GmbH (Vienna, Austria) and cultured in KBMTM-2 basal medium (Lonza, Cat# CC-3103) supplemented with KGMTM-2 SingleQuots TM (Cat# CC-4152; Lonza, Basel, Switzerland) and 50 µg/mL G418 (Cat# ant-gn5; InvivoGen, San Diego, CA, USA).

UVB irradiation: Cells (5 × 10^4^ cells per well in 12-well plates or ×4 × 10^5^ cells in 60 mm dishes) were cultured overnight and irradiated with 50 or 100 mJ/cm^2^ for 14 s and 27 s, respectively, using Medisun PSORI COMB (Schulze & Böhm GmbH, Brühl, Germany). After 24 h, the cell pellets and cell supernatants were collected for analysis.

Talabostat treatment: Cells were cultured with 3 or 30 μM talabostat (Cat# B3941; APExBIO Technology, Houston, TX, USA). After 24 h of incubation, the cell pellets and supernatants were collected for analysis.

### 2.2. Immunoblotting and Antibodies

Proteins from the cell culture supernatants were concentrated using Amicon Ultra centrifugal filters (Cat# UFC500396; Merck, Rahway, NJ, USA). Cells were lysed for 30 min in DISC buffer (30 mM Tris-HCl, pH 7.5, at 21 °C, 120 mM NaCl, 10% glycerol, 1% Triton X-100, and complete protease inhibitor), followed by centrifugation at 14,000× *g* for 10 min to remove cellular debris. Total cellular protein (5–15 µg) was mixed with concentrated Laemmli buffer and boiled at 95 °C for 5 min.

Proteins were separated using NuPAGE Bis-Tris on 4–12% gradient gels (Cat# NP0329BOX; Invitrogen, Waltham, MA, USA) and transferred onto polyvinylidene difluoride membranes. After 2 h of blocking with 5% milk, the membranes were incubated with primary antibodies for IL-1β (dilution 1:2000, AF-201-NAl; R&D Systems, Minneapolis, MN, USA), cleaved IL-1β (dilution 1:1000, #83186; Cell Signaling Technology, MA, USA), caspase 1 (dilution 1:4000, AG-20B-0048; Adipogen, San Diego, Boston, CA, USA), actin (dilution 1:6000, A2103; Sigma-Aldrich), cleaved GSDMD (dilution 1:1000, #36425; Cell Signaling Technology), NLRP1 (dilution 1:1000, #56719; Cell Signaling Technology), cleaved caspase-3 (dilution 1:1000, #9664; Cell Signaling Technology), and ASC (dilution 1:1000, ALX-210-905; Alexis Bio, Boston, MA, USA). Secondary antibodies: Goat anti-mouse IgG1 human ads-HRP (dilution 1:2000, 1070-05), rabbit anti-goat IgG(H+L)-HRP (dilution 1:2000, 6160-05), and goat anti-rabbit IgG-HRP (1:5000, 4030-05), all from SouthernBiotech, Birmingham, AL, USA). Proteins were visualized using Western horseradish peroxidase substrate (Cat# WBLUF0500; Millipore, Burlington, MA, USA). Semi-quantification was performed using ImageJ, 1.54j.

### 2.3. ELISA

IL-1β levels in the supernatants were quantified on a Spectramax 190 microplate reader (Molecular Devices, San Jose, CA, USA) using a Human IL-1 beta/IL-1F2 DuoSet ELISA kit (Cat# DY201; R&D Systems) following the manufacturer’s instructions.

### 2.4. RNA Extraction, Reverse Transcription, and qPCR

Total RNA was extracted using an RNeasy Mini Kit (Cat#74104; QIAGEN, Hamburg, Germany) following the manufacturer’s protocol. Reverse transcription and cDNA synthesis were performed using Superscript III reverse transcriptase (Cat# 18080-044; Thermo Fisher Scientific, Waltham, MA, USA) with a mixture of random nanomers and oligo(dT) primers. qPCR was performed using KAPA SYBR FAST (Cat # KK4617; Merck KGaA). The specific primers for *NLRP1* (For. GCAGTGCTAATGCCCTGGAT; Rev: GAGCTTGGTAGAGGAGTGAGG), *PYCARD* (For. CTTCTACCTGGAGACCTACG; Rev. TATAAAGTGCAGGCCCTGGT, *CASP1* (For. GCCTGTTCCTGTGATGTGGAG; Rev. TGCCCACAGACATTCATACAGTTTC), *IL1B* (For. TTCATTGCTCAAGTGTCTGA; Rev.: TTCATCTGTTTAGGGCCATC), *GSDMD* (For. TTTCACTTTTAGCTCTGGGC; Rev. GACCACTCTCCGGACTAC), and *ACTB* (For. CGCCTTTGCCGATCC; Rev. ACGATCGAGGGGAAGAC) were designed using the NCBI Primer design tool. The following cycling conditions were used: 15 min/95 °C and 42 cycles of 94 °C/15 s, 55 °C/15 s, and 72 °C/30 s. A standard curve was used for the calculation of the qPCR efficiency using the QuantStudio Design&Analysis Software, v1.5.1. Relative quantification of each gene of interest was performed using standard curve values and normalized to the levels of actin as a reference gene.

### 2.5. Propidium Iodide (PI) Staining and FACS Analysis

After treatment, the cells were trypsinized with 0.25% Trypsin–EDTA for 5 min, followed by centrifugation at 200× *g* for 5 min. The cells were resuspended in 500 µL of PBS and stained with 10 µg/mL PI. The FACS analysis was performed using a BD Accuri C6 flow cytometer (BD Biosciences, Franklin Lakes, NJ, USA).

### 2.6. Statistical Analyses

Each ELISA and mRNA expression experiment was performed with three technical replicates per sample. For each diagram, the mean values (±SEM) of two independent experiments are shown.

Each shown WB figure represents one of two to five independent experiments. For mRNA expression experiments, *t*-tests were used for statistical analysis, using basal activity as a control. Statistical analyses were performed using GraphPad Prism 8.

## 3. Results

### 3.1. Expression Profiles of Key Inflammasome Components Varies Among Different Keratinocyte Cell Lines

**Figure 1:**
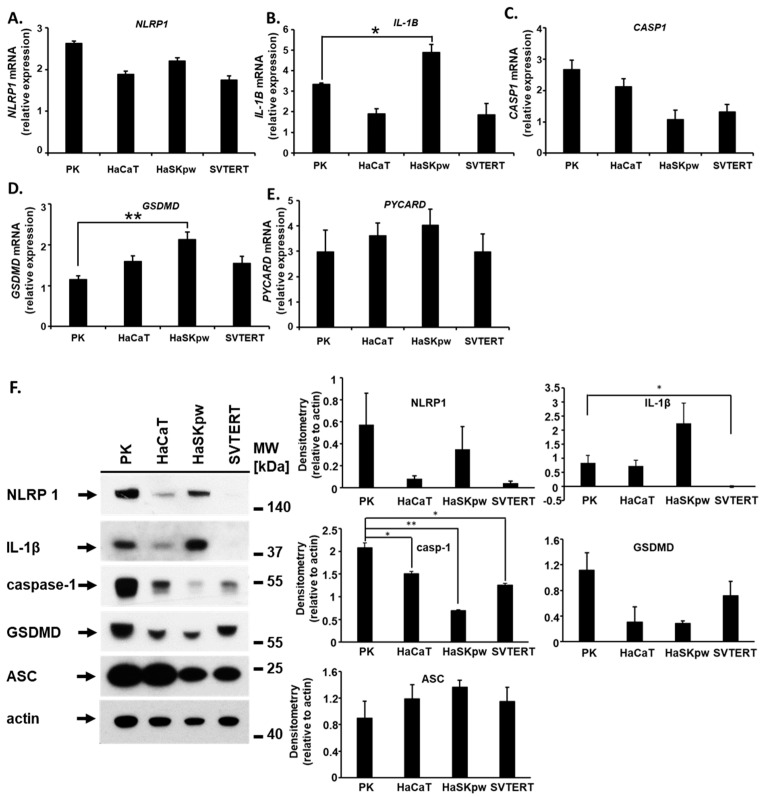
Endogenous expression of the NLRP1 inflammasome components (NLRP1, caspase-1, IL-1β, GSDMD, and ASC) in PKs, HaCaT, HaSKpw, and SVTERT. The indicated cells were cultured until reaching 70% confluence and were harvested for (**A**–**E**) RNA isolation and mRNA analysis and (**F**) Western blotting and semi-quantitative densitometric analysis. Error bars represent the standard deviation (n = 2). * *p* < 0.05; ** *p* < 0.01. Original images can be found in Figure S1.

To compare the expression and function of components of the NLRP1 inflammasome in different keratinocyte cell lines, we first analyzed the expression levels of key inflammasome molecules in HaCaT, HaSKpw, SVTERT, and PK cells at both the mRNA and protein levels. NLRP1 is a broadly expressed molecule with elevated levels in keratinocytes [15,16]. Our mRNA expression analysis revealed a high expression level of *NLRP1* in PKs and lower expression levels in the three immortalized cell lines (Figure 1A). In contrast to *NLRP1*, *IL1B* had more heterogeneous mRNA expression profiles among the cell lines analyzed (Figure 1B). The highest *IL1B* expression was detected in HaSKpw cells, followed by PK cells, which exhibited robust *IL1B* expression. Compared with HaSKpw, the HaCaT and SVTERT cell lines demonstrated more than two-fold lower *IL1B* mRNA levels. *CASP1* mRNA was highly expressed in PKs, followed by HaCaT cells, then HaSKpw and SVTERT cells, both of which showed low expression levels (Figure 1C). In contrast, the expression level of *GSDMD* was highest in the HaSKpw cell line and low in the PK cell line (Figure 1D). All cell lines had similar mRNA expression levels of *PYCARD* (Figure 1E). The results of the protein analysis via Western blotting of the analyzed molecules were compared with the mRNA expression results (Figure 1F). Both NLRP1 and IL-1β proteins were highly expressed in PKs and HaSKpw. In contrast, HaCaT cells demonstrated low expression for both proteins, and neither NLRP1 nor IL-1β were detected in SVTERT (Figure 1F). We found a correlation between the mRNA and protein expression profiles of NLRP1, IL-1β, and caspase-1. In contrast, GSDMD and ASC protein expression levels did not correlate with their mRNA expression levels. Taken together, these data demonstrate that in the analyzed keratinocyte cell lines, key inflammasome components were expressed at varying mRNA and protein levels, which might influence both inflammasome activation and detection in these cells.

### 3.2. UVB Irradiation Activates the Inflammasome in Keratinocytes in a Cell Type-Dependent Manner

**Figure 2:**
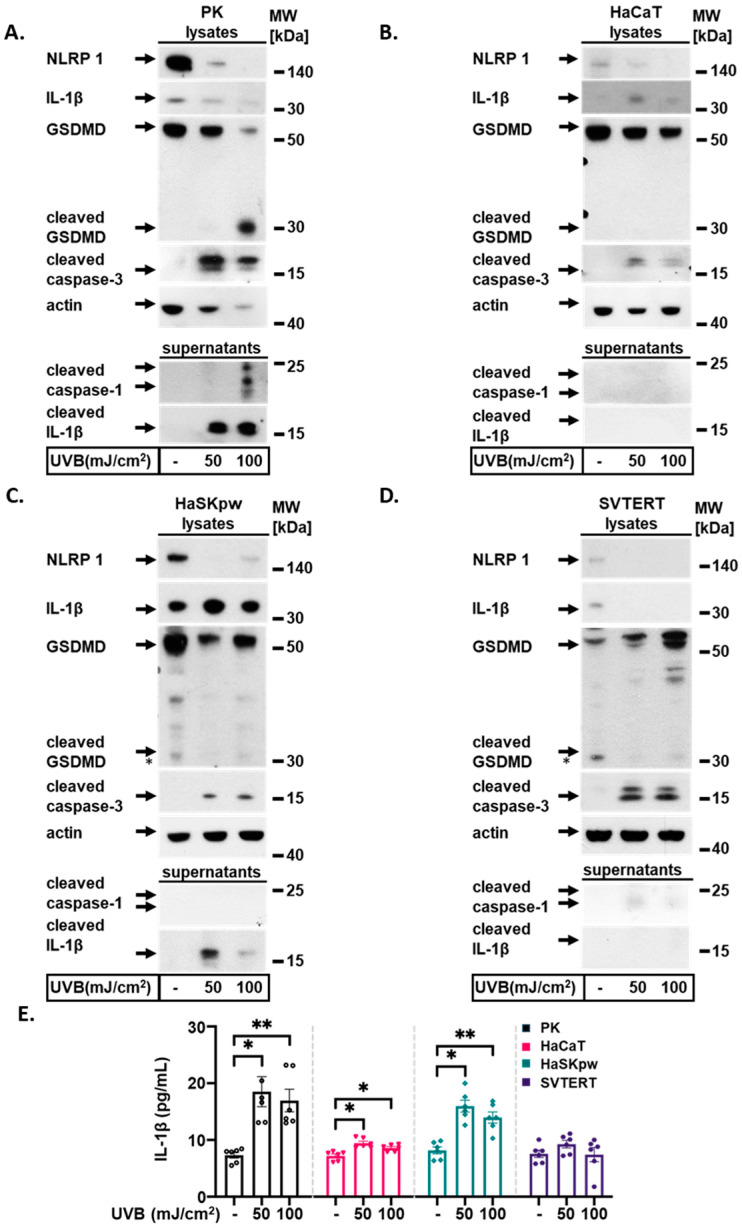
UVB irradiation activates the inflammasome in keratinocytes in a cell type-dependent manner. PK, HaCaT, HaSKpw, and SVTERT cells were UVB irradiated with 50 or 100 mJ/cm^2^ and cultured for 24 h. (**A**–**D**) Western blotting was performed on cell lysates from treated cells to detect the expression of NLRP1, IL-1β, GSDMD, cleaved caspase-3, and on supernatants to detect the expression of cleaved caspase-1 and cleaved IL-1β. (**E**) IL-1β secretion was analyzed using an ELISA. Quantification of the ELISA results from three independent experiments are presented. Error bars represent the SEM. * *p* < 0.05; ** *p* < 0.01. Original images can be found in Figure S2.

Human skin is regularly exposed to sunlight and UVB irradiation. UVB strongly induces NLRP1 inflammasome activation in human keratinocytes [17,18,19]. To assess whether UVB induces NLRP1 activation in a comparable manner in the analyzed cell lines, we irradiated PK, HaCaT, HaSKpw, and SVTERT cells with 50 or 100 mJ/cm^2^. To measure inflammasome activation in irradiated cells, we first performed Western blotting to visualize processed and/or secreted inflammasome proteins in cell lysates and cell supernatants (Figure 2). Protein analyses of irradiated PK cell lysates and supernatants demonstrated strong inflammasome activation measured by a robust secretion of both cleaved IL-1β and cleaved caspase-1 in the supernatant (Figure 2A). Moreover, in the lysates of irradiated PKs, we detected reduced unprocessed IL-1β and the cleavage of GSDMD upon UVB irradiation. These molecules are substrates of active caspase-1. A significant UVB dose-dependent decrease in full-length NLRP1 protein expression was also detected in the lysates of irradiated PKs (Figure 2A). In contrast, no cleaved caspase-1, cleaved IL-1β, or cleaved GSDMD were detected in the cell lysates or supernatants of irradiated HaCaT and SVTERT cells (Figure 2B,D). These results correspond to the low endogenous IL-1β expression in these cells (Figure 1B). As expected, a high level of unprocessed IL-1β was observed in the lysates of HaSKpw cells under control conditions (Figure 2C). However, a UVB dose-dependent decrease in unprocessed IL-1β levels was detected only in the lysates of PKs but not in the lysates of HaSKpw cells (Figure 2A,C), in accordance with the lack of activated caspase-1 in the supernatant of irradiated HaSKpw and the low endogenous caspase-1 expression in this cell line (Figure 1F and Figure 2C). Notably, a signal for processed IL-1β in the HaSKpw supernatant was observed (Figure 2C). Importantly, we detected cleaved caspase-3 in the lysates of all UVB-irradiated keratinocyte cell lines. This was consistent with the known apoptosis-inducing activity of UVB irradiation in human keratinocytes [20]. In PKs and HaSKpw cells, the ELISA revealed a significant and strong increase in secreted IL-1β levels upon UVB irradiation (Figure 2E). In contrast, in HaCaT cells, the increase was significant but relatively weak compared to those in PKs. No significant change in the levels of secreted IL-1β from SVTERT was detected (Figure 2E). These data are partially in line with the Western blotting results and clearly demonstrate that inflammasome activation upon UVB irradiation can be robustly and clearly detected in PKs. In contrast, in the immortalized HaSKpw and HaCaT cell lines, this activation was only partially detected, whereas in the SVTERT cell line, no response to UVB irradiation was observed.

### 3.3. Talabostat Induced NLRP1 Inflammasome Activation Only in Human PKs

**Figure 3:**
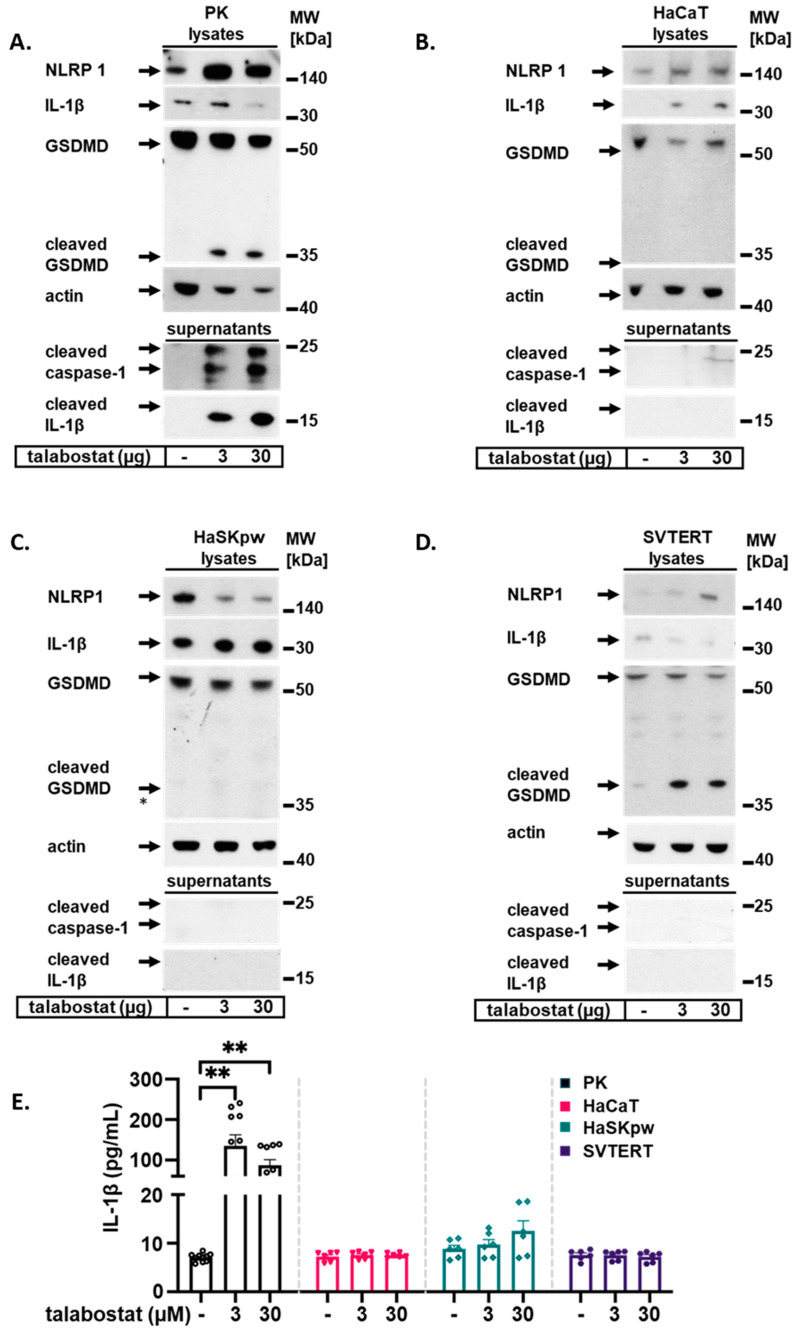
Talabostat induced NLRP1 inflammasome activation only in human PKs. PKs and HaCaT, HaSKpw, and SVTERT cells were treated with talabostat for 24 h. (**A**–**D**) Western blotting was performed on cell lysates from treated cells to detect the expression of NLRP1, IL-1β, GSDMD, and on supernatants to detect the expression of cleaved caspase-1 and cleaved IL-1β. (**E**) IL-1β secretion was analyzed using an ELISA. Quantification of the ELISA results from three independent experiments are presented. Error bars represent the SEM. * *p* < 0.05; ** *p* < 0.01. Original images can be found in Figure S3.

The inhibitor of the DPP8/9 peptidases talabostat is a potent inducer of the NLRP1 inflammasome in PKs [9]. Talabostat induces pyroptosis in human acute myeloid leukemia cell lines but is not cytotoxic in non-acute myeloid leukemia cell lines [21]. Here, we analyzed the effect of talabostat on NLRP1 activation and expression in HaCaT, HaSKpw, and SVTERT keratinocyte cell lines and compared them with those in PKs. We treated all cell lines with two concentrations of talabostat for 24 h and analyzed both cell lysates and supernatants using Western blotting. As expected, both concentrations of talabostat induced strong inflammasome activation in PKs, as confirmed by the detection of cleaved caspase-1 and mature IL-1β in the supernatant of treated cells (Figure 3A). Correspondingly, cleaved GSDMD was detected in the lysates of treated PKs (Figure 3A). Interestingly, an obvious increase in NLRP1 protein expression was noted in PKs upon treatment with talabostat. These data are in line with recently published results that demonstrated a talabostat-dependent increase in NLRP1 protein expression in primary fibroblasts [22]. Similarly, an increase in NLRP1 protein expression was observed in the lysates of HaCaT and SVTERT cells, although the signal was weak (Figure 3B,D). In contrast, talabostat treatment of HaSKpw cells decreased NLRP1 protein expression (Figure 3C). Pro-IL-1β expression decreased in PKs upon talabostat treatment, which was not observed in the other analyzed cells (Figure 3A–D). Importantly, no secreted cleaved caspase-1 or mature IL-1β was detected in the supernatants of any of the talabostat-treated immortalized cell lines (Figure 3A–D), hinting towards a lack of a functional inflammasome in these cells. Correspondingly, the cleavage of GSDMD was detected in PK lysates but not in HaCaT and HaSKpw cell lysates. Notably, cleaved GSDMD was detected in the lysates of SVTERT cells, despite the lack of cleaved caspase-1 (Figure 3D). This discrepancy might be explained by the very low expression level of caspase-1 in SVTERT cells than in PKs (Figure 1B), which could not be detected by Western blotting. Alternatively, another enzyme may be responsible for cleaving GSDMD in SVTERT cells. Additionally, we compared the levels of secreted IL-1β in PKs and the immortalized keratinocyte cell lines using an ELISA (Figure 3E). In line with the results of Western blotting, a robust IL-1β secretion was detected only in the supernatant of PKs, and a slight increase was detected in the supernatant of HaSKpw cells. In contrast, the supernatants of HaCaT and SVTERT cells had a complete lack of secreted IL-1β. Taken together, these data demonstrate that the NLRP1 inflammasome is robustly activated and can be readily detected in PKs but not in the immortalized cell lines tested.

### 3.4. UVB and Talabostat Induce Cell Death in PKs

**Figure 4:**
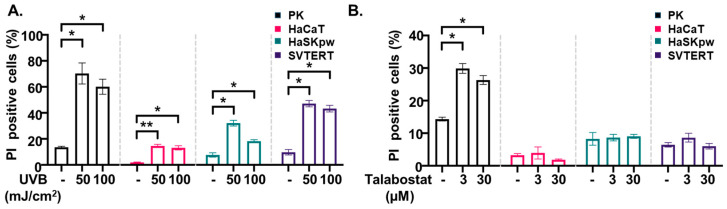
UVB- and talabostat-induced cell death in keratinocytes. PKs, HaCaT, HaSKpw, and SVTERT cells were (**A**) UVB irradiated with 50 or 100 mJ/cm^2^ and cultured for 24 h or (**B**) treated with talabostat for 24 h. Cell death was analyzed using PI staining and FACS analyses. Quantification of the FACS results from three independent experiments are presented. Error bars represent the SEM. * *p* < 0.05; ** *p* < 0.01.

UVB is known to induce cell death in keratinocytes, leading to various forms of cell death such as apoptosis, pyroptosis, and ferroptosis [7,23,24,25,26]. Talabostat-induced cell death has been reported in immune cells [21,27] and to a lesser extent, in keratinocytes [7,9]. To compare the effect of both inflammasome inducers on the sensitivity to cell death, we performed PI staining and FACS analyses of irradiated or talabostat-treated PKs and HaCaT, HaSKpw, and SVTER cells (Figure 4). Our results demonstrate that all keratinocyte cell types were sensitized to cell death upon irradiation with 50 mJ UVB (Figure 4A). However, the susceptibility varied strongly between the cell lines, with a nearly 80% death rate of PKs upon irradiation with 50 mJ, 50% for HaSKpw and HaSKpw cells, and only 10% for HaCaT cells. Correspondingly, in cell lysates from all irradiated keratinocytes analyzed, we detected cleaved caspase-3, a marker of apoptotic cell death (Figure 2A–D). Since the activation of the inflammasome was strongly detected mainly in PKs, the highest sensitivity of primary cells to UVB-induced cell death might be explained by the occurrence of both apoptotic and pyroptotic cell death.

The effect of talabostat on cell viability has been reported primarily in immune cells, such as macrophages and monocytes [9,28,29]. However, PKs are also known to be sensitive to talabostat-induced cell death [9]. Consistently, we observed a significant increase in cell death in PKs upon talabostat treatment in contrast to the immortalized cell lines (Figure 4B).

Altogether, these data suggest that UVB and talabostat can induce inflammasome-related cell death in PKs but not in the immortalized HaCaT, HaSKpw, or SVTERT cell lines.

## 4. Discussion

In skin research, cell culture models were the first means of studying the mechanisms of immunological responses. The choice of an appropriate cell line for the type of study significantly impacts the quality and reliability of the expected results. Primary cells isolated from healthy donors, which preserve the functional properties of the cells, are the closest model system to the original tissue. However, in some experimental settings, the use of PKs can be challenging because of the limitations related to difficulties in genome editing approaches, short lifetimes, and the incapability of the long-term subculturing of primary cells. To overcome these limitations, many researchers have performed studies using cell lines. The advantages of immortalized cell culture include relatively easy handling, higher stability of the cells to changes in the nutrients of growth factors in the media, and a long lifetime. Two spontaneously immortalized cell lines are generally used as two-dimensional in vitro models in skin research, namely HaCaT cells, which are characterized by mutations in the tumor suppressor gene *p53* (*p53*) [30], and HaSKpw cells, which express wild-type *p53* [31]. Additionally, several keratinocyte cell lines have been generated by immortalization through the ectopic expression of human telomerase (hTERT) [32,33], simian virus40 large antigen (SV40) [34], and cyclin-dependent kinase 4 (Cdk4) [35]. SVTERT cells were generated by the immortalization of PKs through the expression of SV40 and human telomerase reverse transcriptase [36,37].

The main aim of this study was to identify the most suitable in vitro model for analyzing inflammasome signaling in keratinocytes by comparing PKs with three immortalized keratinocyte cell lines—HaCaT, HaSKpw, and SVTERT—in terms of inflammasome activation and detection. We compared inflammasome activation upon stimulation with UVB or talabostat by analyzing protein activation and secretion using Western blotting and an ELISA. Additionally, we compared the sensitivity of the analyzed cells to cell death induced by both inflammasome activators using PI staining and FACS.

Our data reveal significant differences between PKs and immortalized keratinocyte cell lines in terms of functional inflammasome activation. Talabostat and UVB irradiation are strong inducers of inflammasome activation in PKs. However, with the exception of the UVB-induced IL-1β cleavage and secretion in HaSKpw cell line, none of the inflammasome inducers initiated clearly detectable inflammasome activation in the analyzed immortalized cell lines. The key molecules needed to form the NLRP1 inflammasome, which are activated by it, are present in variable amounts in cell lines, leading to weaker activation compared with that in primary cells. In the HaSKpw cell line, we detected processed IL-1β upon UVB irradiation but not after talabostat treatment, which partially corresponded to the relatively high expression level of IL-1β in these cells. Both HaCaT and SVTERT cell lines expressed low mRNA and protein levels of NLRP1, caspase-1, and IL-β, which correlates with the undetectable activation of the inflammasome. These data suggest that the expression levels of key molecules in the NLRP1 inflammasome signaling pathway partially correlate with the levels of inflammasome activation and can be used as a marker for detection. Thus, since the amount of endogenous IL-1β is marginal or below the detection level in both HaCaT and SVTERT cells, despite caspase-1 activation, no processed IL-1b is secreted. The differential expression of NLRP1 in PKs and HaSKpw cells compared with HaCaT and SVTERT cells might be based on the different p53 statuses; NLRP1 is regulated in a p53-dependent manner [38].

In summary, we elucidated the differences in NLPR1 inflammasome expression and function between different cell lines and PKs. These findings are essential for identifying an in vitro model suitable for studying inflammasome signaling.

## 5. Conclusions

Based on our data, we conclude that the spontaneously immortalized keratinocyte cell lines HaCaT and HaSKpw, as well as the immortalized keratinocyte cell line SVTERT, are less suitable than primary cells for the analysis of keratinocyte inflammasome signaling pathways. Therefore, despite the advantages in handling and genetic manipulation of immortalized human keratinocyte cell lines, we recommend the use of PKs for analyzing inflammasomes in the skin.

## Data Availability

All data generated in this study are available from the corresponding author upon request.

## Supplementary Materials

The following supporting information can be downloaded at: https://www.mdpi.com/article/10.3390/biom14111427/s1, Figure S1: Original blots to Figure 1; Figure S2: Original blots to Figure 2; Figure S3: Original blots to Figure 3.
