# Supplementary material for: Comparison of Different Keratinocyte Cell Line Models for Analysis of NLRP1 Inflammasome Activation
Source: Biomolecules. 2024 Nov 8;14(11):1427. doi: 10.3390/biom14111427 (PMC11592008; doi:10.3390/biom14111427)
Supplement: Supplementary file 1 [file biomolecules-14-01427-s001.zip › biomolecules-3240298-supplementary.pptx]

## Slide 1
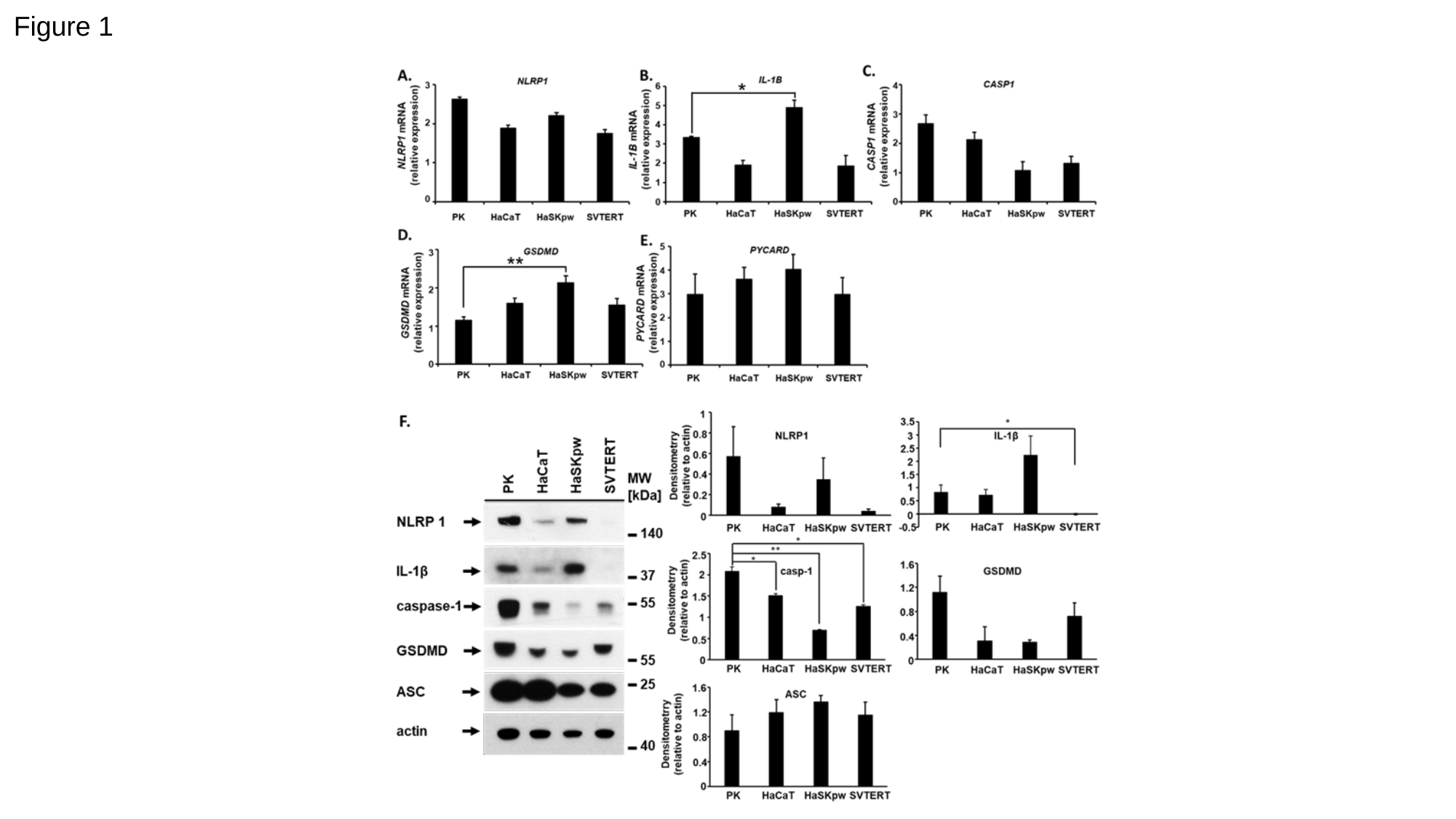

Figure 1

## Slide 2
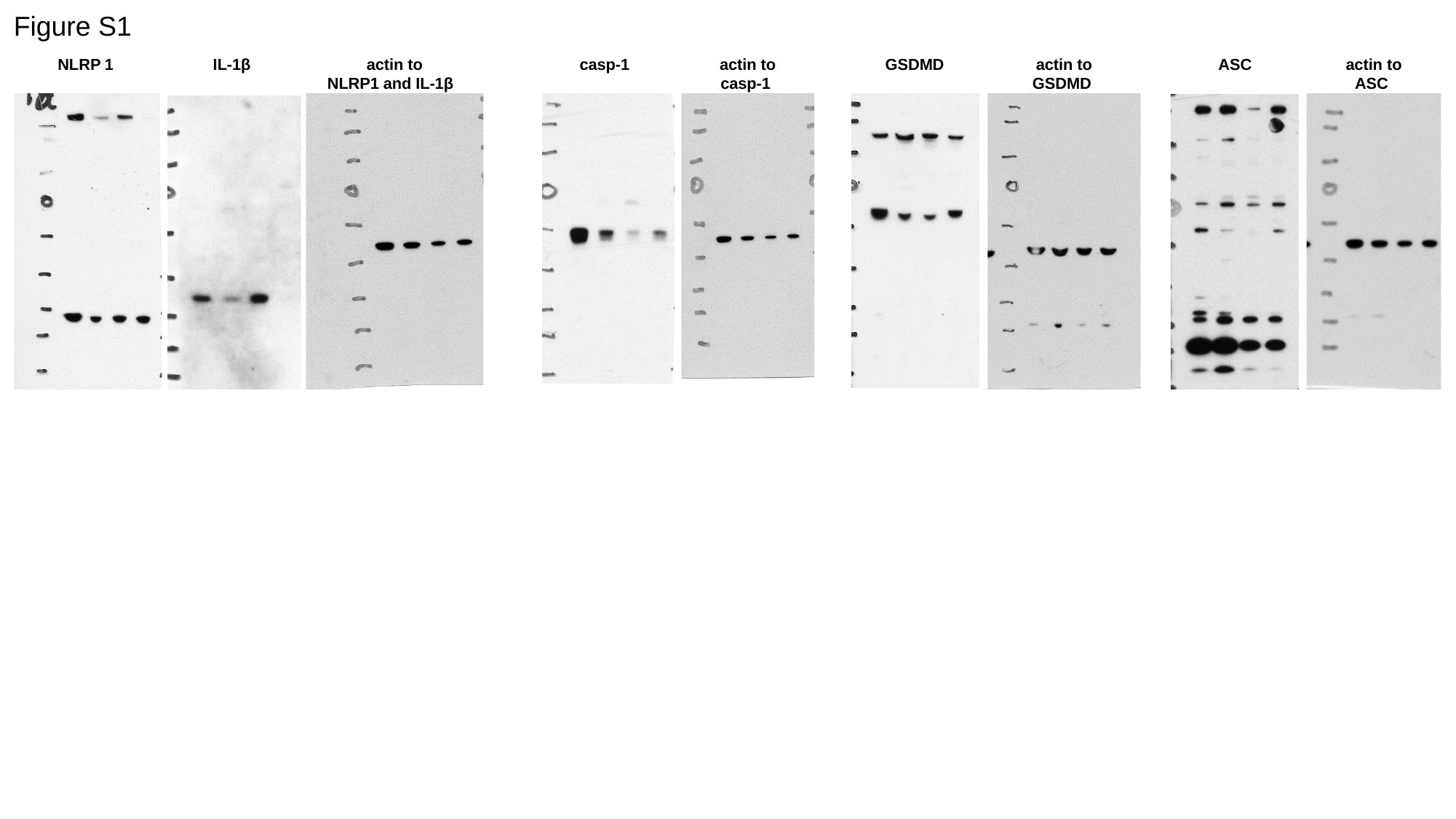

Figure S1
NLRP 1
IL-1β
actin to
 NLRP1 and IL-1β
casp-1
actin to
casp-1
GSDMD
actin to
GSDMD
ASC
actin to
ASC

## Slide 3
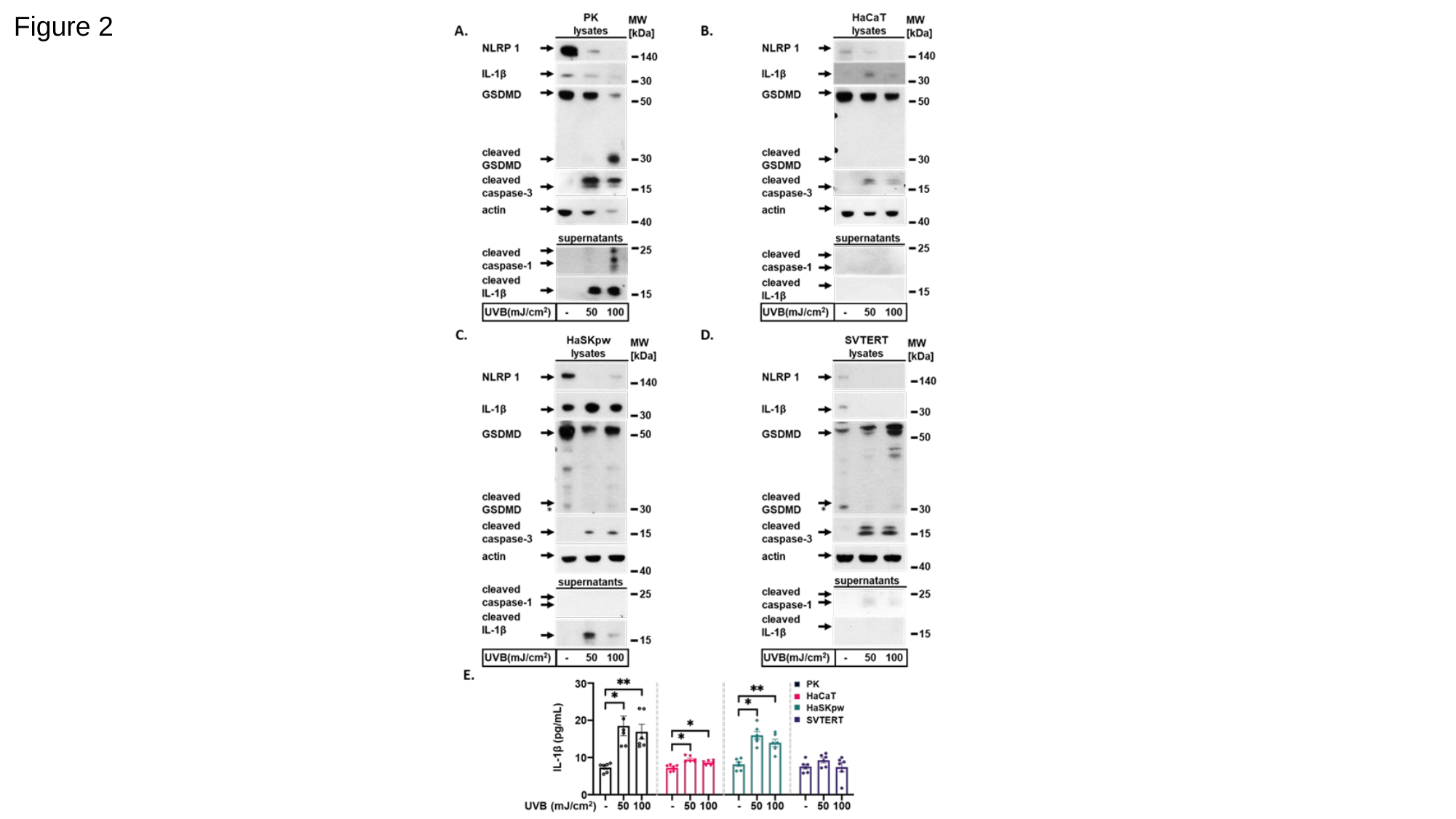

Figure 2

## Slide 4
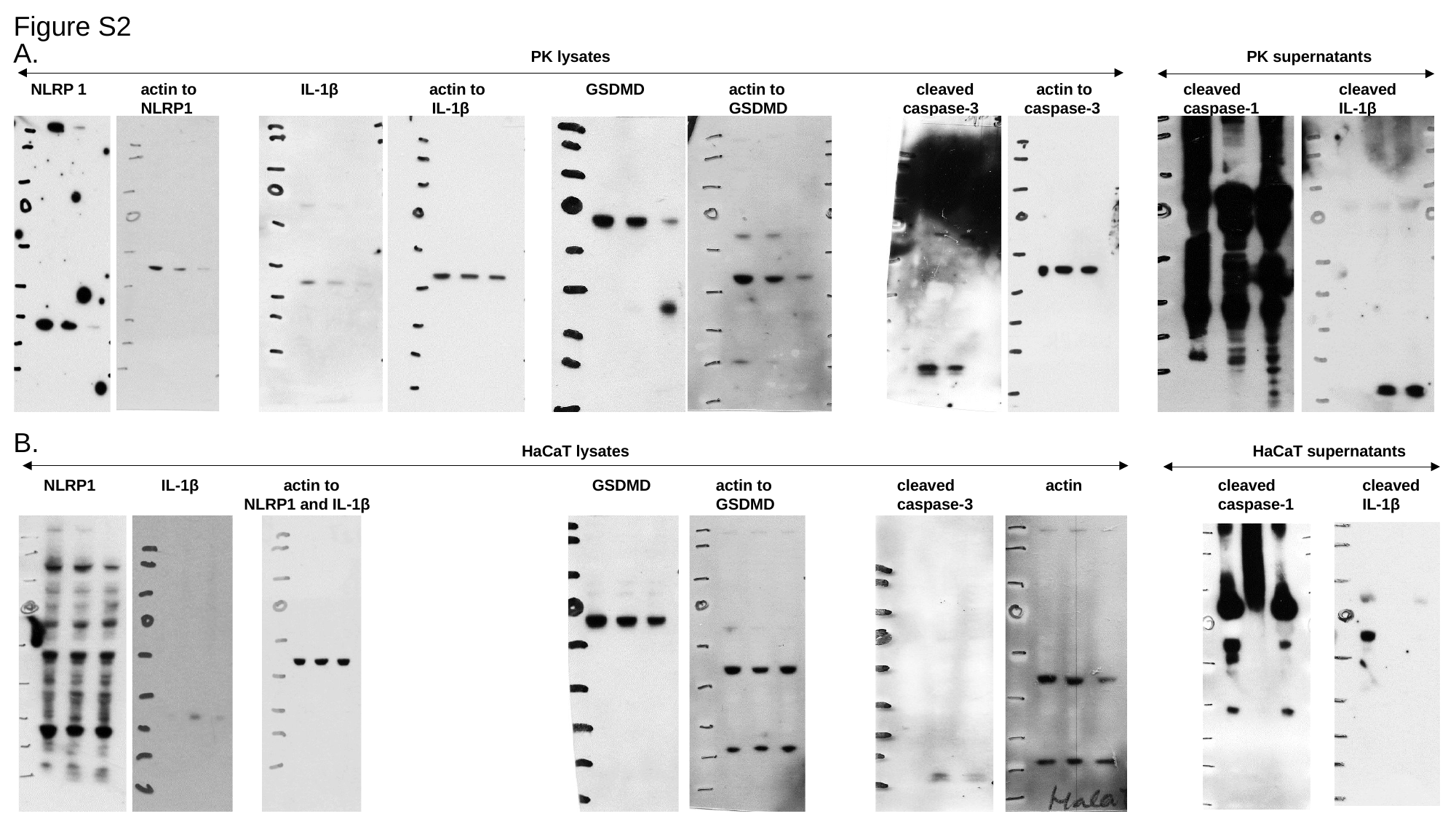

Figure S2
A.
PK lysates
PK supernatants
NLRP 1
actin to
NLRP1
IL-1β
actin to
IL-1β
GSDMD
actin to
GSDMD
cleaved
caspase-3
actin to
caspase-3
cleaved
caspase-1
cleaved
IL-1β
B.
HaCaT lysates
HaCaT supernatants
NLRP1
IL-1β
actin to
 NLRP1 and IL-1β
GSDMD
actin to
GSDMD
cleaved
caspase-3
actin
cleaved
caspase-1
cleaved
IL-1β

## Slide 5
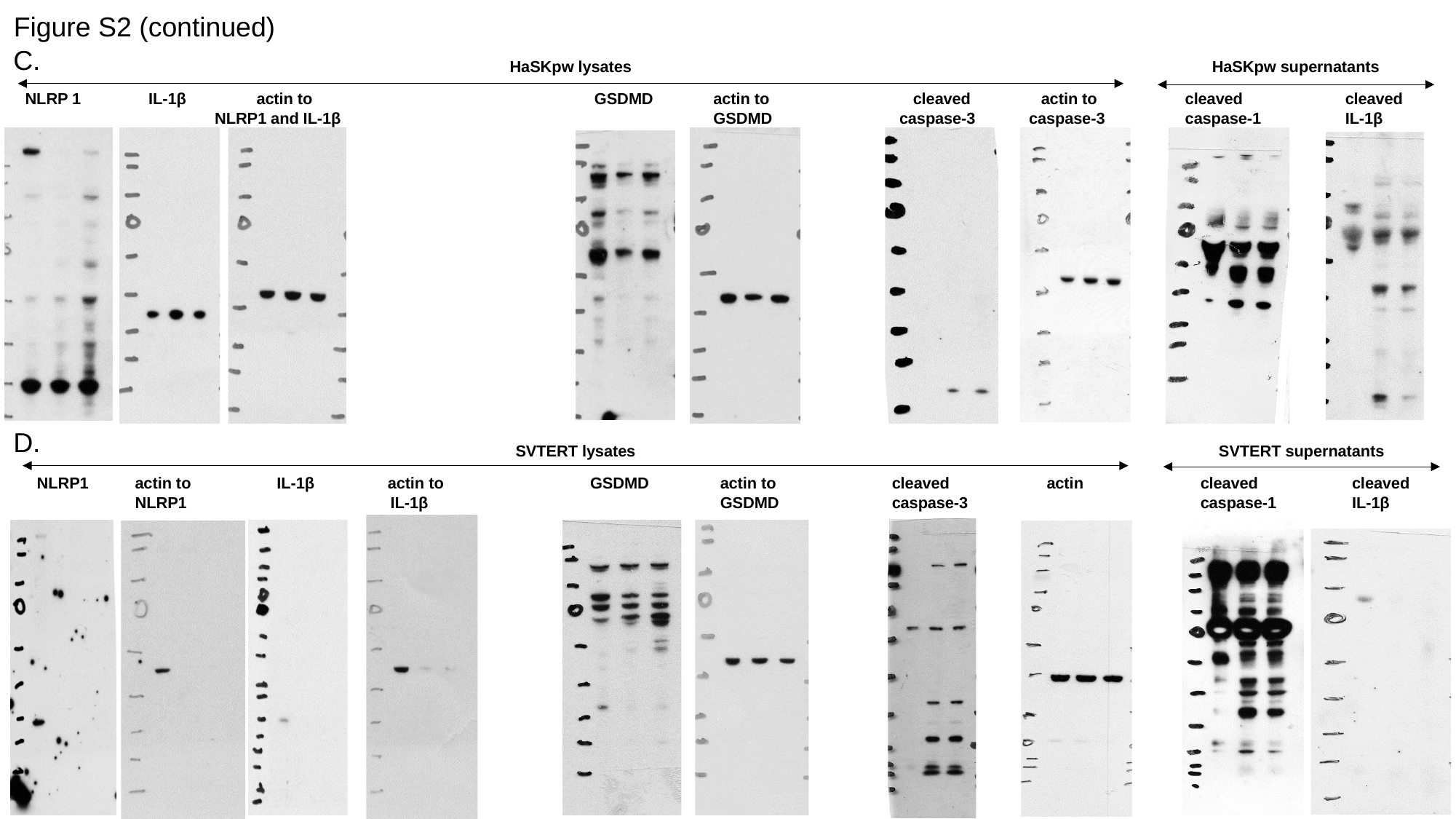

Figure S2 (continued)
C.
HaSKpw lysates
HaSKpw supernatants
NLRP 1
IL-1β
actin to
NLRP1 and IL-1β
GSDMD
actin to
GSDMD
cleaved
caspase-3
actin to
caspase-3
cleaved
caspase-1
cleaved
IL-1β
D.
SVTERT lysates
SVTERT supernatants
NLRP1
actin to
NLRP1
IL-1β
actin to
IL-1β
GSDMD
actin to
GSDMD
cleaved
caspase-3
actin
cleaved
caspase-1
cleaved
IL-1β

## Slide 6
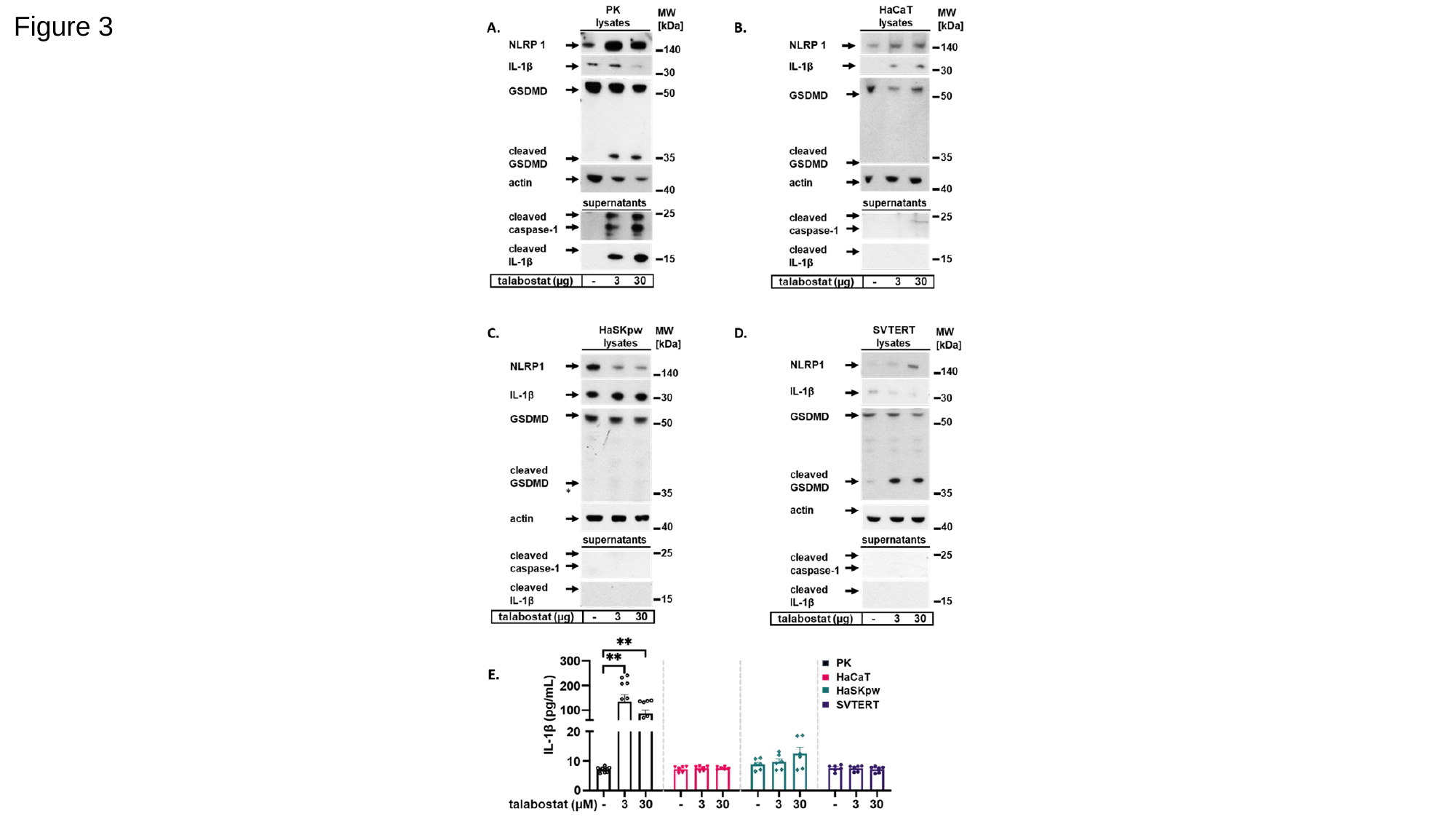

Figure 3

## Slide 7
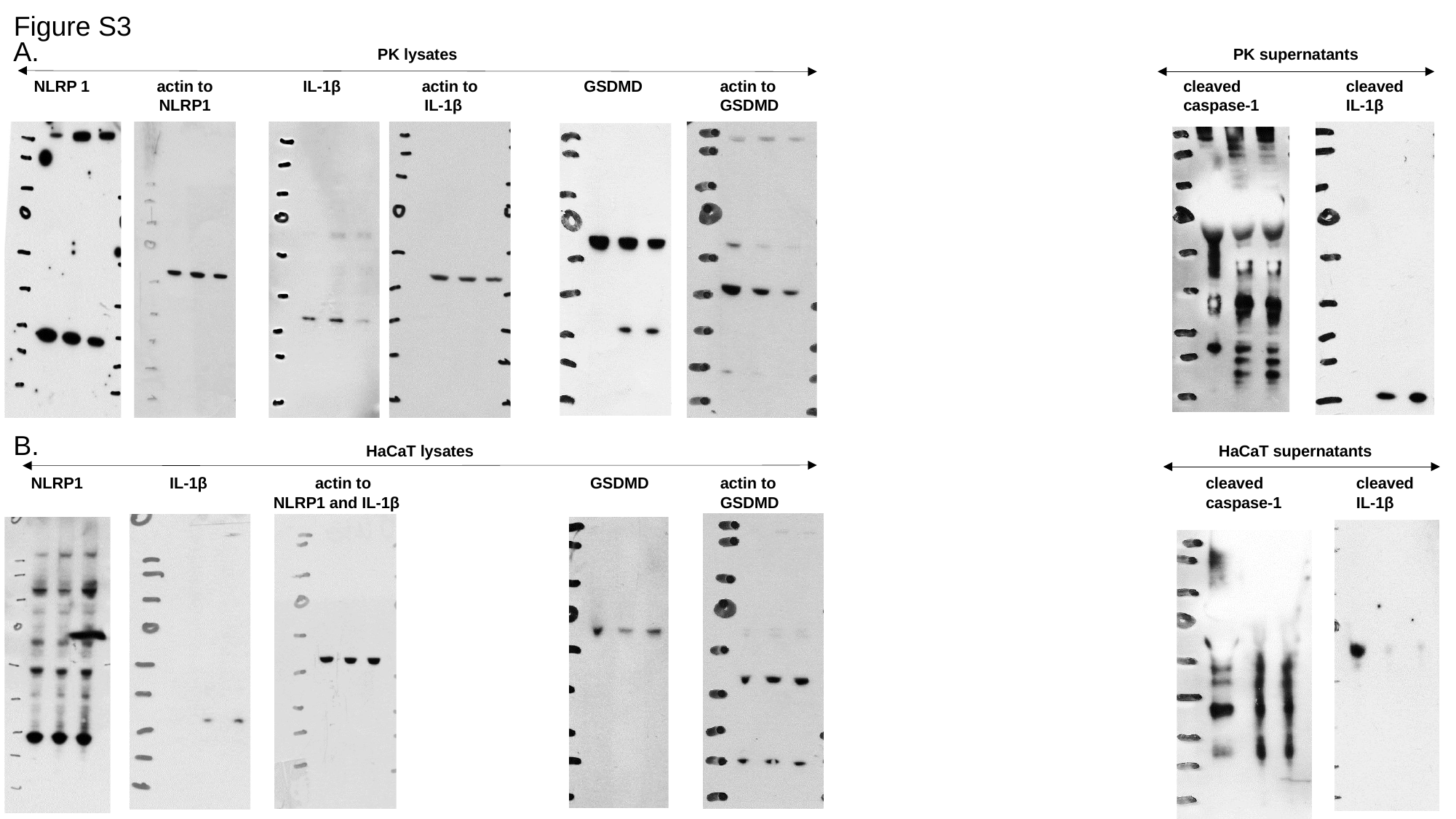

Figure S3
A.
PK lysates
PK supernatants
NLRP 1
actin to
NLRP1
IL-1β
actin to
IL-1β
GSDMD
actin to
GSDMD
cleaved
caspase-1
cleaved
IL-1β
B.
HaCaT lysates
HaCaT supernatants
NLRP1
IL-1β
actin to
NLRP1 and IL-1β
GSDMD
actin to
GSDMD
cleaved
caspase-1
cleaved
IL-1β

## Slide 8
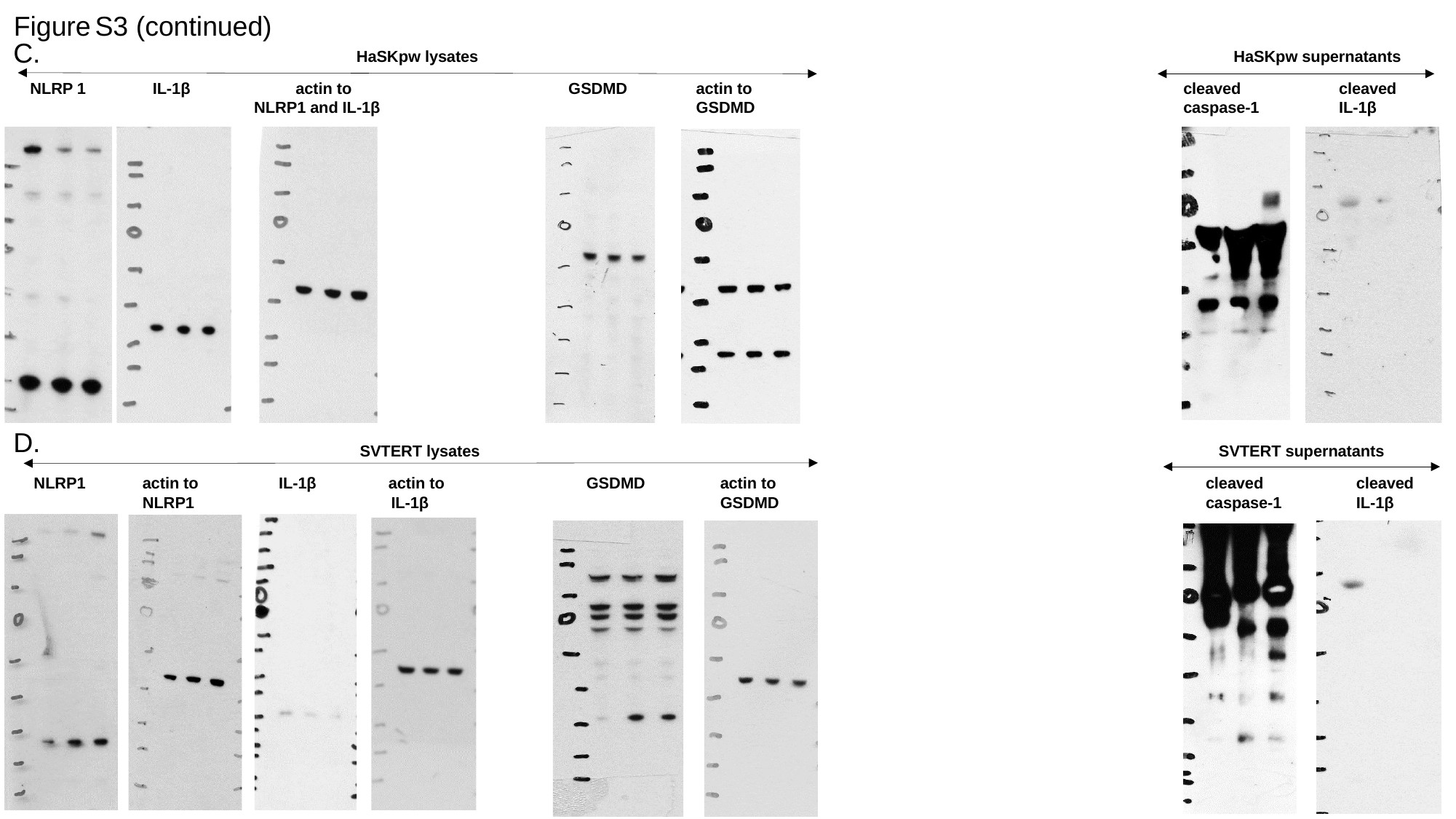

Figure S3 (continued)
C.
HaSKpw lysates
HaSKpw supernatants
NLRP 1
IL-1β
actin to
NLRP1 and IL-1β
GSDMD
actin to
GSDMD
cleaved
caspase-1
cleaved
IL-1β
D.
SVTERT lysates
SVTERT supernatants
NLRP1
actin to
NLRP1
IL-1β
actin to
IL-1β
GSDMD
actin to
GSDMD
cleaved
caspase-1
cleaved
IL-1β

## Slide 9
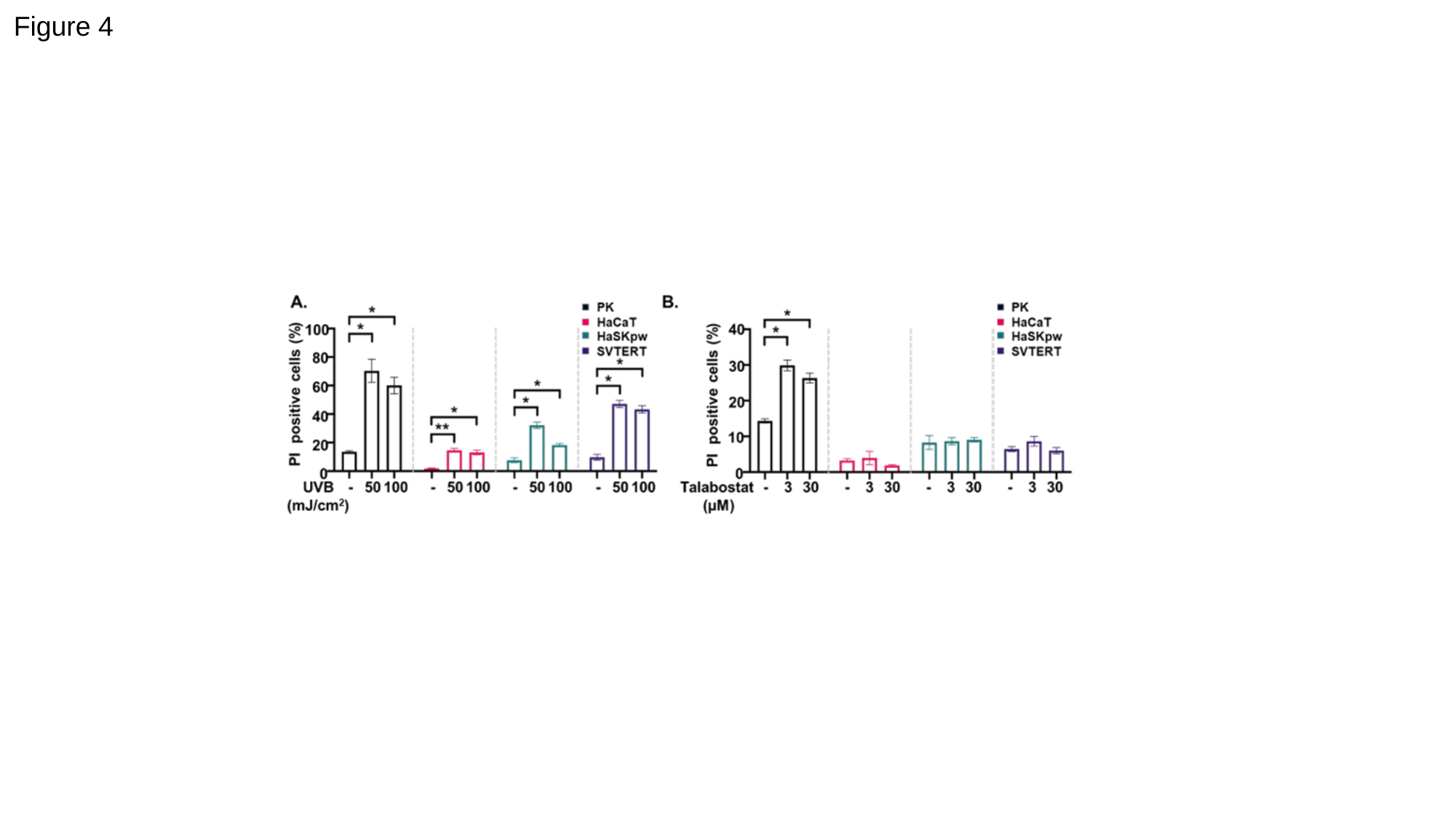

Figure 4
